# Parkinson’s Disease Tremor Detection in the Wild Using Wearable Accelerometers

**DOI:** 10.3390/s20205817

**Published:** 2020-10-14

**Authors:** Rubén San-Segundo, Ada Zhang, Alexander Cebulla, Stanislav Panev, Griffin Tabor, Katelyn Stebbins, Robyn E. Massa, Andrew Whitford, Fernando de la Torre, Jessica Hodgins

**Affiliations:** 1 Center for Information Processing and Telecommunications, Universidad Politécnica de Madrid, 28040 Madrid, Spain; 2 Human Sensing Laboratory, Carnegie Mellon University, Pittsburgh, PA 15213, USA; ajzhang@cs.cmu.edu (A.Z.); alexander.cebulla@kit.edu (A.C.); spanev@cmu.edu (S.P.); Griffin.Tabor@utah.edu (G.T.); stebbinskm@vt.edu (K.S.); whitford@cmu.edu (A.W.); ftorre@cs.cmu.edu (F.d.l.T.); jkh@cs.cmu.edu (J.H.); 3 UPMC Hospitals, Pittsburgh, PA 15213-2582, USA; nadelre@upmc.edu

**Keywords:** in-the-wild supervision, Parkinson’s disease, tremor detection, wearable accelerometers

## Abstract

Continuous in-home monitoring of Parkinson’s Disease (PD) symptoms might allow improvements in assessment of disease progression and treatment effects. As a first step towards this goal, we evaluate the feasibility of a wrist-worn wearable accelerometer system to detect PD tremor in the wild (uncontrolled scenarios). We evaluate the performance of several feature sets and classification algorithms for robust PD tremor detection in laboratory and wild settings. We report results for both laboratory data with accurate labels and wild data with weak labels. The best performance was obtained using a combination of a pre-processing module to extract information from the tremor spectrum (based on non-negative factorization) and a deep neural network for learning relevant features and detecting tremor segments. We show how the proposed method is able to predict patient self-report measures, and we propose a new metric for monitoring PD tremor (i.e., percentage of tremor over long periods of time), which may be easier to estimate the start and end time points of each tremor event while still providing clinically useful information.

## 1. Introduction

Parkinson’s Disease (PD) is a chronic neurodegenerative disorder that can cause a variety of motor symptoms [1]. While these symptoms can be controlled through medication, the long-term use of these drugs can cause side effects, such as dyskinesia (involuntary muscle movements). To limit these side effects, physicians aim to prescribe the minimum dosage necessary to manage the symptoms and periodically adjust the dosage as the disease progresses. Physicians typically measure disease severity by using the Unified Parkinson’s Disease Rating Scale (UPDRS) [2], but the UPDRS is inherently subjective and is only conducted every three to six months, when patients meet with their physician. Patient diaries allow patients to record information about their PD state between clinic visits [3]. However, frequently recording detailed entries is burdensome, and symptoms can be difficult to recall accurately.

**Figure 1:**
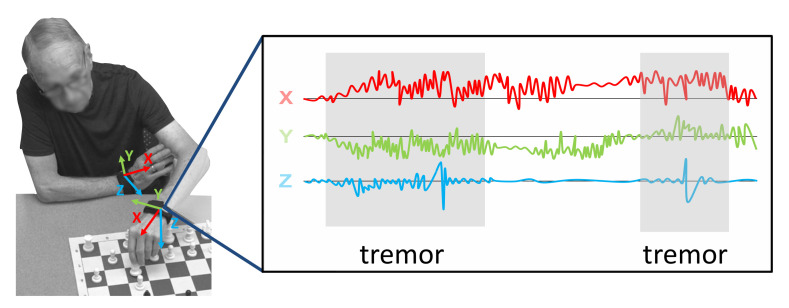
Automated tremor detection with wearable sensors during everyday activities.

A better way to track PD symptoms would be a fully autonomous system capable of continuously monitoring and analyzing motions during everyday life (Figure 1). With no need for input from the patient, such a system could have high temporal resolution and low subjectivity. However, many challenges remain in developing algorithms for automated PD symptom detection. For example, it is difficult to distinguish PD symptoms, such as tremor, from normal activities of daily living, such as brushing teeth. Furthermore, there is high variability between patients, both in their symptom manifestations and daily activities. Robust feature sets and machine learning (ML) algorithms should be able to perform with high accuracy despite this variability.

Additional challenges arise when aiming to maintain high symptom detection performance in natural living environments (i.e., in the wild). Labels of symptom events are typically obtained by referencing video data in laboratory data collections. In wild settings, however, video data are generally not available, meaning that accurate labels (exact start and end of each symptom) cannot be obtained. At best, weak labels (presence, absence, or the approximate amount of a symptom within a time segment) can be provided by participants of the data collections. Weakly supervised algorithms, such as multiple-instance learning, explicitly account for weak labels, and tend to perform better than standard, fully supervised learning algorithms in these scenarios [4]. In summary, collecting data, labeling them, and learning from such data are all more challenging in wild environments. However, such work is necessary because, as established by [5], the performance of algorithms trained on laboratory data does not necessarily translate to wild data.

In this work, we analyze several feature sets and algorithms for PD tremor detection in both laboratory and wild conditions. In particular, we compare a standard baseline feature set and four new feature extraction methods, some inspired by traditional speech processing techniques and others learned automatically through Convolutional Neural Networks (CNNs). Each feature set is followed with either a Random Forest (RF) or Multi-Layer Perceptron (MLP) classifier to distinguish the various performance contributions of features versus algorithms. We compare the performance of our best feature set and algorithm combination with the three most relevant systems described in the literature: [4,6,7]. We also compare our method with [4] (best among [6,7]) in the ability to reproduce patient self-reports, the current standard for in-home symptom monitoring. Finally, we propose a new metric for monitoring PD tremor (percentage of tremor time measured over longer periods), which is easier to detect than specific tremor events. This new metric provides information about the symptom prevalence, and it could be used by the physician to adjust the dosage of the medication. Note that this work focuses on symptom detection, as opposed to severity estimation, to reduce the scope of the problem of learning in the wild. Additionally, of the many PD symptoms, PD tremor is chosen because motor symptoms are easier to detect with wearable devices, and tremor is the most prevalent PD motor symptom [8].

## 2. Related Work

Many researchers have explored the use of machine learning to automatically detect PD motor symptoms with wearable sensor [9,10,11], considering several motor symptoms at the same time in several cases [12]. Despite this strong interest, there are no commercial systems that provide a good user experience or sufficient accuracy to complement doctor assessments. Several challenges remain, such as overall accuracy in daily life, clinically meaningful metrics, and detection of symptoms in a new subject for whom there are no training data. This section reviews related work on different feature sets, classification algorithms, and in-the-wild monitoring systems in general.

### 2.1. Feature Sets

A large number of feature sets have been proposed for PD detection. The vast majority rely on time domain features (such as the mean, range, or cross-correlation) [13,14], frequency domain features (such as the dominant frequency, energy content in a particular band, or signal entropy) [15,16], or a combination of the two [17,18,19,20]. Some authors have shown that features that are traditionally used for speech processing (e.g., Mel frequency, Cepstral coefficients) are also effective for classifying human motion from accelerometer data [21,22]. In this work, we evaluate whether these speech processing features can improve PD tremor detection accuracy. Furthermore, given the strong performance of CNNs in a variety of applications, we also evaluate the performance of features learned by a CNN for tremor detection.

### 2.2. Classification Algorithms

Researchers have experimented with a wide variety of standard ML algorithms, such as decision trees [14], support vector machines (SVMs) [17], random forests (RFs) [23], hidden Markov models [15], and dynamic neural networks [13]. Some studies compared several of these algorithms [24]. More recently, researchers have explored the efficacy of deep learning techniques for automated UPDRS testing [25] or bradykinesia detection [26,27]. In this work, we evaluate the performance of a full CNN architecture for tremor detection, as well as that of a Multi-Layer Perceptron (MLP) when hand-crafted features are used.

### 2.3. In-the-Wild Monitoring Systems

There is substantial literature on systems to explore PD symptom detection. However, the majority of related work evaluates and compares algorithm performance on data collected and labeled in laboratory settings. A smaller body of work specifically addresses symptom detection in the wild.

Zhan et al. [28] presented a smartphone-based monitoring platform that measures PD symptoms actively (i.e., data recorded during specific tests at certain times during the day) and passively (i.e., data recorded continuously in the background). The target of this work was not to monitor PD symptoms, but to discriminate treatment from baseline in order to monitor medication response remotely. Only the data recorded during specific tests were used for this analysis. Labels were generated by clinic visits at home on a weekly basis for three months.

Lipsmeier et al. [29] used a smartphone app to collect data from Parkinson participants and healthy controls during active motor tests (performed six times daily). The smartphone was also used during the day (passive monitoring) to compute the time spent walking and sit-to-stand transitions. The goal was to obtain digital biomarkers calculating the correlation between some features and the MDS-UPDRS (Movement Disorders Society UPDRS) values obtained every two weeks.

Both [28] and [29] required participants to perform specific activities multiple times per day; symptom severity (MDS-UPDRS value) was then estimated from data collected during these activities. However, performing these tests several times per day can be burdensome for patients, leading to issues in compliance. PD monitoring through passive, continuous monitoring in the wild where the system automatically detects symptoms could have a better acceptance. However, neither [28] nor [29] collected labels of symptom occurrences, making it difficult to use such data to train an algorithm for continuous monitoring. Indeed, in both studies, passive data were only used to separate Parkinson’s patients from healthy controls. This paper focuses on continuous PD tremor detection under natural living conditions, without requiring users to behave in any specific way. Algorithms for this application need labeled data collected in the wild for training and validation.

Kinesia^TM^, developed by Great Lakes NeuroTechnologies Inc., is a commercial system for continuous tremor monitoring. However, detailed results on in-home PD tremor detection have not been published. In an in-home study for essential tremor detection [30], the algorithm output was correlated with their automated tremor assessment test [31], but not compared to a human-labeled ground truth. More recently, the group has published results for continuous assessment of levadopa’s response by monitoring PD symptoms in laboratory settings [6].

Researchers at the University of Newcastle have collected data in laboratory and home settings [7,32]. Data from all patients were mixed together and then split into training and testing sets using either seven-fold or leave-one-day-out cross-validation. Note that including data from a test patient during training does not provide person-independent results.

Das et al. [19] is one of the first to use weakly supervised learning algorithms on weakly labeled in-home data. However, the in-home dataset was very small, with only two PD patients, and hence did not allow for leave-one-subject-out cross-validation (LOSO). This work was followed by Zhang et al. [4], where multiple weakly supervised learning algorithms were compared on a larger dataset collected in a laboratory setting under LOSO. The authors also proposed a “stratified” modification to multiple-instance learning, which demonstrated higher performance than standard multiple-instance learning in the context of PD tremor detection. In [5], the authors compared the performance of several algorithms on laboratory and wild data. The results indicated that performance can differ significantly between laboratory and wild data, highlighting the importance of training and validating on wild data for applications where algorithms are expected to perform in the wild.

Heijmans et al. [33] include a study for tremor detection using wearable sensors during daily life. The proposed method compared the information collected from subjects’ questionnaires (weak labels) using an Experience Sampling Method (ESM) app named PsyMate^TM^, with objective metrics computed from inertial signals: logarithmic signal energy between 3.5 and 7.5 Hz, root mean square of the low-pass-filtered (3 Hz) time series, dominant frequency and dominant energy ratio, amplitude range of the raw time series, and maximum normalized cross-correlation and corresponding temporal offset between all accelerometer and gyroscope channels. An Area Under the Curve (AUC) value of 73% was reported.

Papadopoulos et al. [34,35] proposed a method for tremor detection using in-the-wild recordings from a smartphone. This method included a multiple-instance learning approach for training a deep neural network. This study obtained very good detection results, but the system was evaluated considering postural tremor (the subject was holding the smartphone during a phone call). Other types of tremors, like kinetic tremor, are more difficult to detect because tremors are mixed with subjects’ movements.

In this work, we propose several new algorithms and feature sets, comparing them to previous literature on both laboratory and wild data.

## 3. Methods

**Figure 2:**
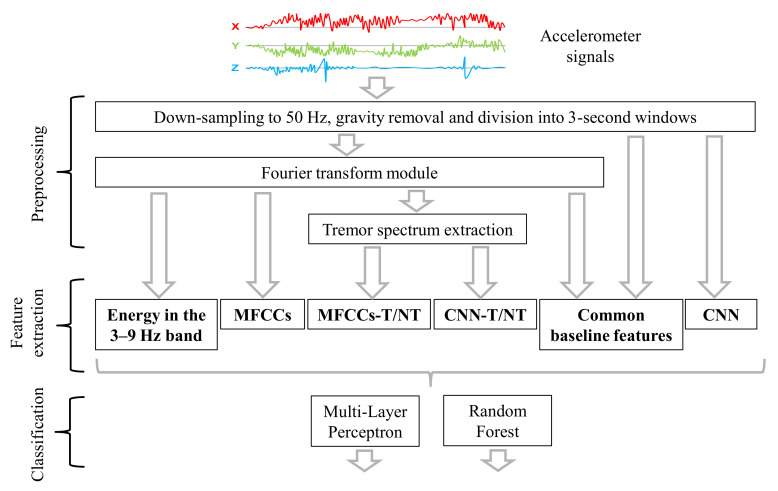
Diagram of the computation framework.

This section describes the gathered dataset, signal pre-processing steps, six proposed feature sets, and our tremor detection algorithms. We consider both fully labeled and weakly supervised data. Figure 2 shows a diagram of the different feature sets and classification algorithms that are evaluated in the paper.

### 3.1. Data Collection

Our dataset includes laboratory and in-the-wild three-axis (*X*, *Y* and *Z*) recordings from Axivity AX3 accelerometers positioned on both wrists with a sampling rate of 100 Hz. Both data collections were approved by the Carnegie Mellon University Institutional Review Board in accordance with the Helsinki Declaration.

#### 3.1.1. Laboratory Recordings (LAB)

We collected accelerometer data from 12 participants, aged 62–85 years, who were diagnosed 2–5 years prior with PD. Each participant performed various activities of daily living, such as making a sandwich, writing, typing, and playing chess and cards, and completed several motor tasks taken from Part III of the UPDRS [2]. We recorded frontal, left-side, and right-side views of each session to minimize occlusion of both hands. Ground-truth labels for the presence or absence of tremor in the accelerometer signals were obtained by manually segmenting periods of time with and without tremor in both arms independently. This labeling process was carried out by referencing the video recordings. The same person segmented all the data from all participants to ensure label consistency. This person was specifically trained to segment tremor periods using the information from video cameras. These ground-truth labels were used to train the machine learning algorithms. The participants were selected because they reported tremor (although some did not show tremor during the session). Table 1 shows the percentage of tremor time during laboratory sessions. Participants 1 and 6 did not exhibit any tremor during the data collection and were thus excluded from this study. This dataset is referred to as LAB data. Additionally, the motor tasks were evaluated by a medical expert according to the UPDRS (see Table 2). This table includes evaluation of rest tremor (when the muscle is relaxed), postural tremor (when holding a pose), kinetic tremor (when performing some actions or movements), and specific movements like finger tapping, hand movements, and pronation/supination movements. Note that, as this paper focuses on detection and not severity estimation, the UPDRS scores were not used for training. However, they are provided to show the presence of other symptoms in order to give a better sense of the classification challenge for this specific dataset. Furthermore, they will be used to discuss the results in the experimental validation.

#### 3.1.2. In-the-Wild Recordings (WILD)

Participants 2, 4, 5, 10, 11, and 12 agreed to wear two wrist-worn accelerometers throughout the day for four weeks. Labels of tremors were provided by the participants. In order to promote frequent labeling throughout the day, participants submitted labels through a cell phone app, which was designed to prevent participants from submitting many entries within a short time span or backdating entries. The app prompted participants to submit an entry roughly every hour, and participants were paid per entry. To improve label accuracy, participants were only asked to record the amount of tremor they experienced within the five minutes prior to submitting the entry. Following the recommendation given in previous work [4], we provided three label options (*Almost none*, *Half the time*, and *Almost always*). We name them weak labels because they have information about the presence/absence of tremor in a long segment of times but the exact temporal location of the tremor is unknown. All participants made regular entries during the four weeks, submitting roughly 300 entries each. This dataset with the weak labels is referred to as WILD data.

### 3.2. Preprocessing

This section describes the pre-processing steps (i.e., downsampling, filtering, and windowing) that are common to all feature sets. For all feature sets, we downsampled the raw accelerometer signals to 50 Hz in the time domain. Information loss was negligible because the energy in the frequency spectrum above 25 Hz was less than 1% of the total energy. Furthermore, the energy of normal human movement is between 0 and 3 Hz, and the energy of tremor primarily falls between 3 and 9 Hz [36]. To remove the influence of gravity, we filtered the data with a high-pass third-order Butterworth filter at 0.3 Hz. Afterwards, the sample sequence was divided into three-second windows (150 samples per window) with a two-second overlap. A window was labeled as a tremor if more than 50% of it overlapped with a tremor event.

#### Non-Negative Tremor Factorization

For some feature sets, in order to enhance the tremor signal, we developed a method to extract information of the tremor spectrum from the total spectrum before feature extraction. This method is performed in an unsupervised manner, without using any labels. This method was inspired by the voice extraction method proposed by Durrieu et al. [37], in the context of speech processing, which uses non-negative matrix factorization (NMF).

We assume that the amplitude spectrum of every window $S^{\mathrm{Total}}$ can be estimated by ${\hat{S}}^{\mathrm{Total}}$, which is decomposed into a tremor spectrum $S^{\mathrm{Tremor}}$ and a non-tremor spectrum $S^{\mathrm{Non}-\mathrm{tremor}}$:

(1) $${\hat{S}}^{\mathrm{Total}}=S^{\mathrm{Tremor}}+S^{\mathrm{Non}-\mathrm{tremor}}.$$

Similarly to Durrieu et al. [37], we decompose the tremor spectrum into an excitation spectrum $S^{\mathrm{Source}}$ modulated (i.e., multiplied) by a spectral shaping envelope $S^{\mathrm{Shape}}$. Both terms are modeled as a linear combination of frequency functions, $t_{i}\left(f\right)$ and $h_{i}\left(f\right)$, respectively:

(2) $$S^{\mathrm{Tremor}}=S^{\mathrm{Source}}•S^{\mathrm{Shape}},$$

(3) $$=\sum_{i=1}^{N}w_{i}^{t}t_{i}(f)•\sum_{j=1}^{M}w_{j}^{h}h_{j}(f),$$

where • indicates element-wise multiplication, *N* and *M* are the number of filters, and $w_{i}^{t}$ and $w_{j}^{h}$ represent the weights for frequency functions $t_{i}\left(f\right)$ and $h_{i}\left(f\right)$. The number and type of filters are based on Durrieu et al.’s approach [37].

**Figure 3:**
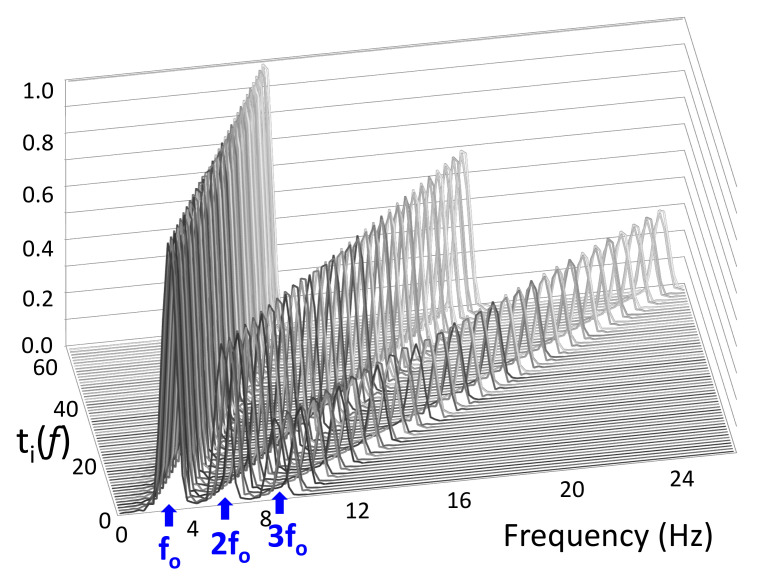
T(f) matrix. Rows are ideal tremor spectra, which each consist of a base frequency $f_{0}$ and two integer harmonics ($2f_{0}$ and $3f_{0}$). Base frequencies are set every 0.1 Hz from 3–9 Hz.

The matrix $T\left(f\right)={\left[t_{1}\left(f\right),t_{2}\left(f\right),\ldots ,t_{N}\left(f\right),1\right]}^{⊤}$ consists of N=60 “ideal” tremor spectra, $t_{i}\left(f\right)$, with base frequencies set every 0.1 Hz from 3–9 Hz, and a vector of ones for modeling windows without any movement (Figure 3). The ideal tremor spectra are generated by combining three *sinc* functions situated at $f_{0}$ (tremor base frequency), $2f_{0}$, and $3f_{0}$. This design is informed by the frequency spectrum of tremor, where peaks occur not only in the standard 3–9 Hz range, but also at their two corresponding harmonics. We considered two harmonics because the third tremor harmonic does not appear in our dataset.

The matrix $H\left(f\right)={\left[h_{1}\left(f\right),h_{2}\left(f\right),\ldots ,h_{M}\left(f\right)\right]}^{⊤}$ consists of M=63 Hann’s functions $h_{i}\left(f\right)$, which have a width of five points and are equally spread across the frequency domain with 50% overlap. Note that representing the tremor spectrum shape as a weighted sum of Hann’s functions reduces the number of variables that need to be estimated.

The non-tremor spectrum is modeled as follows:

(4) $$S^{\mathrm{Non}–\mathrm{tremor}}=\sum_{i=1}^{M}w_{i}^{a}a_{i}\left(f\right),$$

where each $a_{i}\left(f\right)$ is equal to $h_{i}\left(f\right)$).

In order to estimate the different weights,

$$\begin{array}{cc} w^{t} & ={\left[w_{1}^{t},w_{2}^{t},\ldots ,w_{N}^{t},w_{N+1}^{t}\right]}^{⊤},\\ w^{h} & ={\left[w_{1}^{h},w_{2}^{h},\ldots ,w_{M}^{h}\right]}^{⊤},\;\mathrm{and}\\ w^{a} & ={\left[w_{1}^{a},w_{2}^{a},\ldots ,w_{M}^{a}\right]}^{⊤}, \end{array}$$

we solve a non-negative least-squares problem with Euclidean distance,

(5) $$\min_{w^{t}w^{h}w^{a}}∥S^{\mathrm{Total}}-{\hat{S}}^{\mathrm{Total}}{∥}_{2}^{2},$$

where ${\hat{S}}^{\mathrm{Total}}=w^{t}T\left(f\right)\times w^{h}H\left(f\right)+w^{a}A\left(f\right))$.

The algorithm to estimate the weights uses the update rule from NMF [38]:

(6) $$w_{\mathrm{new}}^{t}=w^{t}•\frac{(S^{\mathrm{Shape}}•S^{\mathrm{Total}})T{\left(f\right)}^{⊤}}{(S^{\mathrm{Shape}}•{\hat{S}}^{\mathrm{Total}})T{\left(f\right)}^{⊤}}$$

(7) $$w_{\mathrm{new}}^{h}=w^{h}•\frac{(S^{\mathrm{Source}}•S^{\mathrm{Total}})H{\left(f\right)}^{⊤}}{(S^{\mathrm{Shape}}•{\hat{S}}^{\mathrm{Total}})H{\left(f\right)}^{⊤}}$$

(8) $$w_{\mathrm{new}}^{a}=w^{a}•\frac{S^{\mathrm{Total}}A{\left(f\right)}^{⊤}}{{\hat{S}}^{\mathrm{Total}}A{\left(f\right)}^{⊤}},$$

where $A(f)={\left[a_{1}\left(f\right),a_{2}\left(f\right),\ldots ,a_{M}\left(f\right)\right]}^{⊤}$. ${\hat{S}}^{\mathrm{Total}}$ must be recomputed after every update. After the last iteration, the weight $w_{N+1}^{t}$, which is associated with the all-ones row of T(f), is considered a threshold for the rest of the weights $w_{i}^{t}$, and all weights $w_{i}^{t}$ under this threshold are set to zero. Using a validation set, we found that, typically, 100 iterations led to sufficient convergence of the weight vectors.

**Figure 4:**
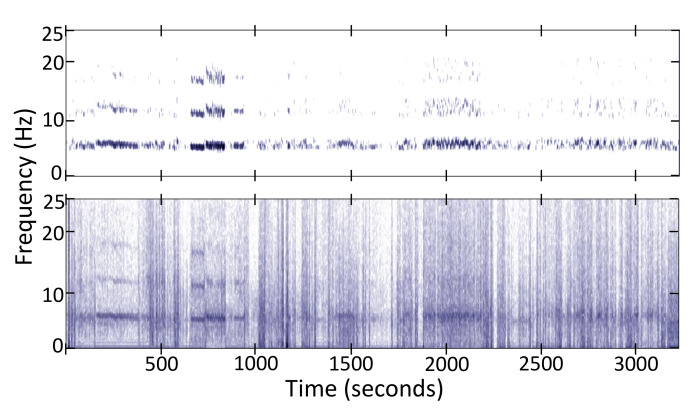
Tremor spectrum (**Top**) extracted from the total spectrum (**Bottom**) of the *z*-axis acceleration signal.

Given data from a particular participant, we can compute the average power spectrum for all windows. This computation allows us to personalize this general tremor spectrum extraction procedure in two ways: (1) We multiply the 60 ideal tremor spectra (T(f)) with the average spectrum shape for this participant to emphasize the tremor spectrum that best fits the participant’s tremor. (2) We estimate the base tremor frequency $f_{0}$ by detecting a peak in the 4–8 Hz interval of the average spectrum shape and then constrain the weights $w^{t}$ so that only the three maximum weights within a ±1 Hz interval around $f_{0}$ are considered. The rest of the weights are set to zero. Figure 4 shows an example spectrum from raw data and the tremor information extracted from it.

### 3.3. Feature Extraction

This paper evaluates seven different feature sets.

#### 3.3.1. Energy in the 3–9 Hz Band (Energy Threshold)

PD tremor energy falls mainly between 3 and 9 Hz [36], and a simple threshold on the energy in this band serves as a basic benchmark. In order to represent the Receiver Operating Characteristic (ROC) curve, we evaluated the system in a range of thresholds between the minimum and the maximum 3–9 Hz energy observed in the training set.

#### 3.3.2. Welch’s One-Sided Power Spectral Density (PSD)

We considered Welch’s one-sided Power Spectral Density (PSD). The PSD is represented with 128 points between 0 and 25 Hz for each axis.

#### 3.3.3. Common Baseline Features (Baseline)

In the time domain, we extracted 16 basic features; the number in parenthesis indicates the dimension. We compute mean (1), standard deviation (1), median (1), max (1), min (1), signal magnitude area (1), energy (1), inter-quartile range (2), empirical cumulative distribution function (4), entropy (1), and auto-regression coefficients (2). In the frequency domain, we calculated the module of the Fast Fourier Transform (FFT) (spectrum), and from this spectrum, we computed the same 16 features described above, and an additional 11 features: dominant frequency (1), average frequency (1), spectral power (1), skewness (1), kurtosis (1), and the energy of six equally spaced frequency bands (6). In the frequency domain, we extracted a total of 27 features (16 + 11).

The 16 time-domain features and 27 frequency domain features are computed on the *X*-, *Y*-, and *Z*-axes, as well as the magnitude vector of both acceleration and jerk (signal derivative). In addition, we computed the correlation coefficient between every pair of axes for the acceleration, jerk, FFT of acceleration, and FFT of jerk signals, leading to 12 correlation coefficients. In total, the baseline feature set included 356 features: (16 + 27) (features) × 4 (axes: X, Y, Z and magnitude) × 2 (signals: acceleration and jerk) + 12 (correlation coefficients).

#### 3.3.4. Mel Frequency Cepstral Coefficients (MFCCs)

As commented above, we divided the signal into three-second windows with a two-second overlap using a Hamming window. For each axis, we compute 12 MFCCs, leading to a feature set of 36 MFCCs.

#### 3.3.5. Mel Frequency Cepstral Coefficients after Tremor Spectrum Extraction (MFCCs-T/NT)

We computed 12 MFCCs on the original signal and the two spectra obtained using the tremor spectrum extraction method described in Section 3.2. In total, we computed 36 MFCCs per axis, or 108 MFCCs in total for each three-second window.

#### 3.3.6. Features Learned with a CNN Trained on Raw Data (CNN)

**Figure 5:**
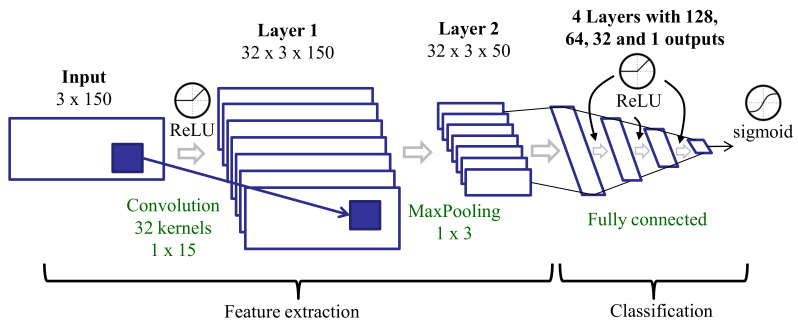
Deep learning architecture for training on raw data.

We trained a CNN (Figure 5) on the raw data. The CNN was made up of several layers organized into two parts: The first part learned the main features from the inputs and included one convolutional layer with a max-pooling layer, and the second part integrated four fully connected layers for classification. The convolutional layer took as input the raw time domain signal of each three-second window. Considering a 50 Hz sampling rate, every three-second window included 150 samples for each axis, *X*, *Y*, and *Z*. The input dimensions were 3 (axes) × 150 (samples in a three-second window). The convolution layer applied 1D filters independently along each axis. The padding parameter of this layer was set to *same*, resulting in an output with the same dimensions as the input. The outputs were then combined using a max-pooling strategy. The outputs of the max-pooling layer were the features considered in this approach. The number and size of the kernels were defined considering a compromise between the number of parameters in the CNN and the amount of data available to train them.

The activation function was ReLU (Rectified Linear Unit) in all layers except the last one (output), where a sigmoid was used. With ReLU functions, there is a lower probability of gradient vanishing (the gradient has a constant value). The training process used the binary-cross-entropy loss function and the *rmsprop* optimizer with a batch size of 50. In this optimization method, each unit kept its own mean gradient feedback throughout the learning process. At each step, the given gradient value was normalized by this mean, avoiding oscillating weight updates. The weights of the convolutional layer were initialized using a simple autoencoder with three layers (initial, encoding, and decoding). The weights from the encoding layer were used to initialize the convolutional layer. The rest of the layers in the CNN were initialized randomly using a Glorot uniform initializer.

There were dropout layers (deactivating 20% of the weights) after convolutional and full connected layers to avoid over-fitting during the training process. The number of epochs in every experiment was adjusted using a validation subset extracted from the training set.

#### 3.3.7. Features Learned with a CNN Trained on Spectra after Tremor Spectrum Extraction (CNN-T/NT)

**Figure 6:**
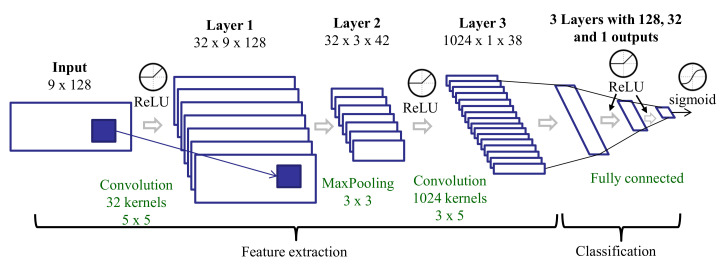
Deep learning architecture for training on spectra after tremor spectrum extraction.

This feature set is similar to the CNN features (see Section 3.3.6) with a slightly different deep learning architecture. The CNN includes two convolutional layers, and the features are the outputs of the second convolutional layer. The input to the CNN includes the original spectrum of each three-second window and the two spectra from the tremor extraction preprocessing: tremor and non-tremor spectra (see Section 3.2). As there are three axes, we have nine spectra for every three-second window. Each spectrum is represented using 128 points in the 0–25 Hz frequency range. These spectra are stacked to create a 2D input (9 × 128) to the network (Figure 6). These spectra were obtained after a Hamming windowing. The number of layers and their dimensions differ slightly in this architecture compared to that trained on raw data, but all other aspects (activation functions, training process, etc.) are the same.

### 3.4. Classification Algorithms

This section describes two different classifiers, and evaluates their performance in two scenarios: (1) fully supervised learning on LAB data (accurate labels) and (2) weakly supervised learning with a multiple-instance learning (MIL) algorithm on WILD data (weak labels).

#### 3.4.1. Fully Supervised Learning

For fully supervised learning, we compare two classification algorithms. We use a random forest (RF) (with 100 decision trees) as our baseline algorithm. RF achieves comparative results to other traditional ML algorithms, such as a decision tree, *k*-nearest neighbor, or an SVM. Our second algorithm is a Multi-Layer Perceptron (MLP) because they are typically put at the end of deep architectures for final classification. Our MLP is constructed of three fully connected layers with 128, 32, and 1 neurons, respectively. The activation function is ReLU in all layers except the last one, where a sigmoid is used.

#### 3.4.2. Weakly Supervised Learning

To train on weak labels, we used the MIL algorithm proposed by Zhang et al. [4]. This algorithm iterates through three steps: First, the system predicts the class of every three-second window contained in a five-minute interval. These predictions, which are output from a sigmoid layer, can be thought of as pseudo-probabilities. Next, all three-second windows in every five-minute interval are sorted according to these probabilities. Finally, a subset of the windows are selected from each five-minute interval for retraining. In particular, if the weak label of a five-minute interval is *Almost none* or *Almost always*, the bottom (least likely to be tremor) 50% or top (most likely to be tremor) 50% of the windows within that interval are selected for retraining, respectively. For intervals labeled as *Half the time*, the bottom 25% and top 25% of the windows are selected. The algorithm terminates when it reaches a set number of iterations, chosen using a validation subset selected from the training set. Note that, in the first iteration, no predictions are available for sorting the windows because the system is yet to be trained. Therefore, to initialize this algorithm, the three-second windows are sorted according to the estimated energy of the tremor spectrum, which is computed in an unsupervised manner using the non-negative tremor factorization described in Section 3.2. With this initialization, the number of iterations is lower than 10.

## 4. Experiments

This section describes the experimental validation, which addresses four main questions:

1. How well do our proposed feature sets and ML algorithms generalize to patients that are not in the training set?
2. How does our best system (feature set + ML algorithm) compare to previous work?
3. How well can an automatic method reproduce patient self-assessments of tremor, the current standard for in-home monitoring?
4. How well can an automated system approximate the percentage of tremor time over long intervals (hours, days, or weeks)?

Unless otherwise noted, all experiments use leave-one-subject-out (LOSO) cross-validation to assess performance: classifiers are trained using data from every subject but one, and performance is measured by testing on the data from the held-out subject. This approach simulates a realistic scenario where there are no training labels for a new patient.

### 4.1. Evaluating Performance on Lab Data

**Figure 7:**
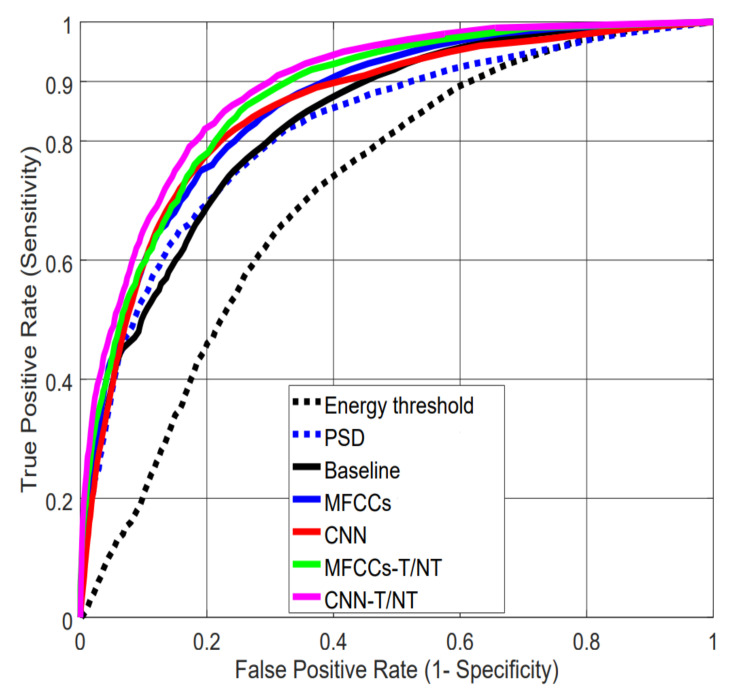
Receiver Operating Characteristic (ROC) curves for the different feature extraction strategies using the MLP classifier. Note: The ROC curve for the “energy in the 3–9 Hz band” feature is computed by sliding the threshold over the range of possible values.

Table 3 summarizes the Area Under the Curve (AUC) values and False Positive Rates (FPRs) at a 0.90 True Positive Rate (TPR) for each pairing of the seven feature sets and two classification algorithms. The results are the average over all the subjects using a leave-one-subject-out cross-validation. Figure 7 shows the Receiver Operating Characteristic (ROC) curves for all feature sets using an MLP for classification. With this dataset, a difference of 0.01 in AUC is significant with *p*-value < 0.0001, according to Hanley’s method [39].

The results in Table 3 indicate that feature sets, not algorithms, drive performance: AUC values are similar between RF and MLP within a particular feature set, but differ across feature sets. We can see that the simple baseline of thresholding energy performs much worse than the more complex feature sets. In particular, the results show the importance of tremor spectrum extraction (-T/NT): Not only do MFCCs and CNNs show significant improvement when computed after this preprocessing step compared with raw data, but CNN-extracted features computed on raw data (CNN) perform worse than our handcrafted MFCCs computed after tremor spectrum extraction (MFCCs-T/NT).

**Figure 8:**
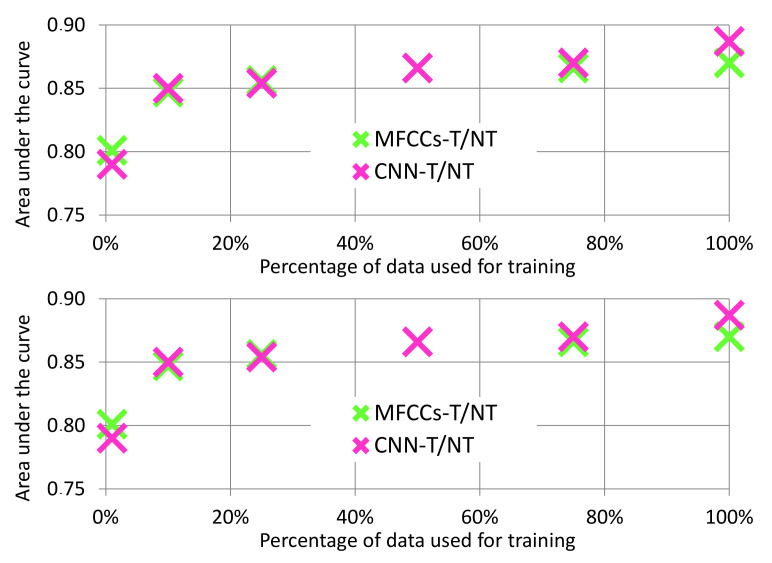
Analysis of AUC values over varying amounts of training data. (**Top**): Data reduced for feature learning only. Mel Frequency Cepstral Coefficients after Tremor Spectrum Extraction (MFCCs-T/NT) features are not learned, which means they are independent of dataset size. (**Bottom**): Data reduced for both feature learning and MLP training.

The CNN-T/NT features had the highest performance, but the performance from the MFCCs-T/NT features was close. Furthermore, because the CNN-T/NT features are learned using labeled data, the quality of these features relies on having access to relatively large datasets. In contrast, the MFCCs-T/NT features are not learned from data and, hence, are independent of the dataset size. Figure 8 (top) depicts how the CNN-T/NT features degrade when given less data: CNN-T/NT performance drops below that of MFCCs-T/NT when <50% of the training data are available. Note that the training data are used only for the feature extraction process and not for the classification algorithm: We train the convolutional layers using a small amount of data, fix the weights, and then train the MLP using the full dataset.

While the previous experiment demonstrates the sensitivity of the CNN-T/NT features to the size of the training dataset, training part of a deep network on a small dataset and training the rest of it in a larger dataset is not a realistic scenario. Figure 8 (bottom) shows the performance of both feature sets when data are also reduced for the MLP classifier. We can see that when <50% of the training data are available, performance of the two systems is similar. This finding implies that, while the CNN-T/NT features perform better with our dataset, our handcrafted MFCCs-T/NT features may be useful when small datasets are all that is available.

### 4.2. Comparison to Previous Work on LAB Data

**Figure 9:**
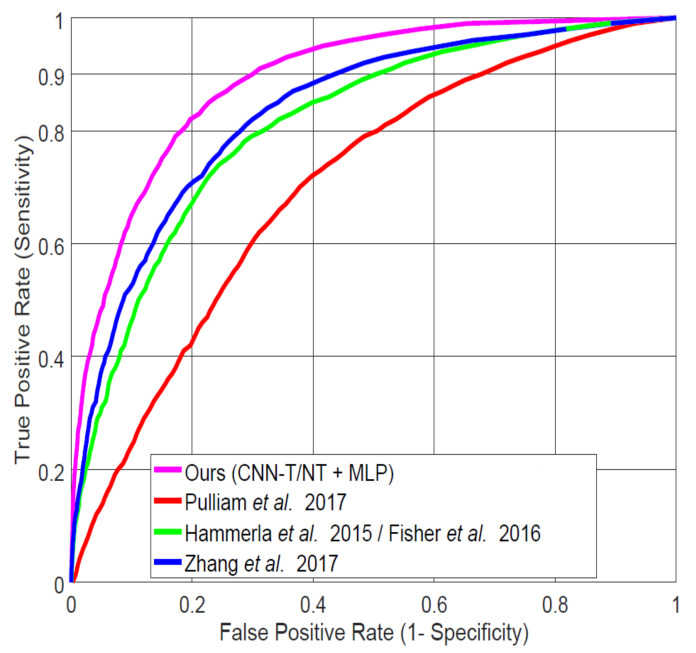
Comparing our best system (Convolutional Neural Network Trained on Spectra after Tremor Spectrum Extraction (CNN-T/NT) + MLP) on lab-recorded (LAB) data to previous systems proposed in the literature using leave-one-subject-out (LOSO) cross-validation.

In order to compare our best-performing system (CNN-T/NT + MLP) with published studies for in-home data, we implemented the systems of Pulliam et al. [6] (details described in [40]), Hammerla et al. [7] (also used by Fisher et al. [32]), and the best-performing system analyzed by Zhang et al. [4]. Figure 9 shows the ROC curves for each of the systems applied to our LAB data, and Table 4 specifies AUC and FPR at 0.9 TPR values. Our system demonstrates significant improvement over those from the previous studies. All subsequent experiments compare our CNN-T/NT + MLP system with the next best one (Zhang et al. [4]).

### 4.3. Reproducing Tremor Self-Assessments in Patients’ Diaries

The current standard for in-home monitoring of PD relies on patient self-assessments of their symptoms. Here, we perform five different experiments to analyze how well our algorithms can replicate this current standard by computing Spearman’s correlation coefficient [41] between our system’s output and the weak labels (*Almost none*, *Half the time*, and *Almost always*) that were assigned to the five-minute intervals by our participants using the app. To convert our instance-based system output into the corresponding weak label, we use the percentage of detected tremor in the five-minute interval, assigning <33%, 33–66%, and >66% tremor to *Almost none*, *Half the time*, and *Almost always*, respectively. We evaluated the system considering different types of training and testing sets in order to perform a detailed analysis.

#### 4.3.1. LAB/WeakLAB

The system is trained on accurate labels from the LAB data and tested on synthetically generated weak labels from the LAB data. The synthetically generated labels are generated using the accurate labels by computing the percentage of tremor in five-minute intervals (with a four-minute overlap): *Almost none* (<33% tremor), *Half the time* (33–66%), and *Almost always* (>66%). Because these labels are accurately generated, this experiment represents an upper limit on the accuracy measured as a correlation coefficient.

#### 4.3.2. LAB/WILD

The system is trained on accurate labels from the LAB data and tested on weak labels from the WILD data. This experiment estimates performance of the system in a realistic scenario: trained on data collected in a laboratory setting, but expected to classify data collected in a user’s home.

#### 4.3.3. WeakLAB/WILD

The system is trained on LAB data with synthetically generated weak labels. Because accurate labels for the WILD data do not exist, this experiment serves as a fair comparison of training on the LAB data versus WILD data.

#### 4.3.4. WILD/WILD

The system is trained and tested on weak labels from the WILD data. This experiment estimates the effect of training on data collected in natural settings.

#### 4.3.5. WeakLAB + WILD/WILD

The system is trained simultaneously on (weakly labeled) LAB and WILD data to benefit from the reliability of weak labels from LAB data, but also from the greater variability of the WILD data.

**Figure 10:**
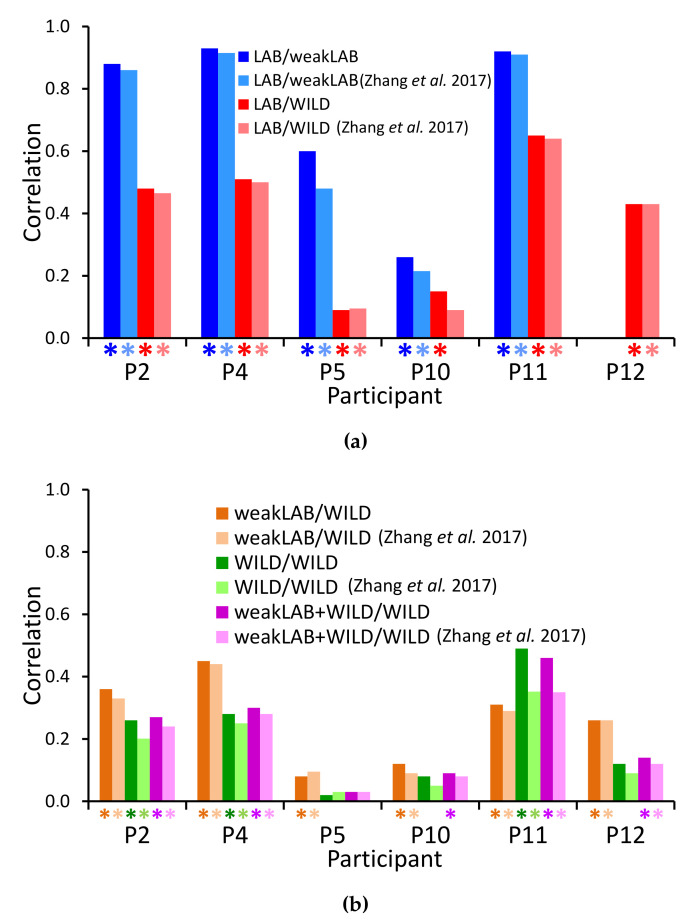
Spearman’s correlation coefficients between the classifier output and the assigned labels. Asterisks represent correlations for which the null hypothesis of no correlation can be rejected with *p* < 0.005. (**a**) Training on accurately labeled data (LAB only), testing on LAB or in-the-wild (WILD) data. Note: Participant 12 was excluded from the LAB/weakLAB experiment due to insufficient tremor. (**b**) Training on weakly labeled data (LAB, WILD, or both), testing on weakly labeled WILD data.

Figure 10 shows the correlation values between the weak labels and the outputs from either CNN-T/NT + MLP or Zhang et al. [4] in the five experiments. Note that random guessing would lead to a correlation of 0. Almost all correlations are significant, i.e., the null hypothesis of no correlation can be rejected with p<0.005. Correlations are higher for participants 2, 4, and 11. These participants showed high percentages of tremor time during the lab session (see Table 1) and high scores in the MDS-UPDRS scale for resting tremor (see Table 2). Participant 5 only showed postural and kinetic tremor with low percentages of tremor time during the session. Participant 10 did not show tremor very often during the LAB session (12% of the session for the right hand, and 8% for the left hand) with predominance of resting tremor. Participant 12 showed very little tremor during the LAB session (1% of the session for the right hand, and 2% for the left hand) with predominance of resting tremor. Although our system, in general, obtains slightly higher correlations with the weak labels than that of Zhang et al. [4], the differences are not significant.

All decreases in correlation from LAB/weakLAB to LAB/WILD experiments are significant (Figure 10a). The lower performance is unsurprising because it is harder to detect tremor in everyday activities than in laboratory conditions with prescribed activities, and there is likely more variability in the labels in the wild. Furthermore, when computing the correlation on WILD data, we assume that all weak labels are accurate. In contrast to the WILD data, weak labels from the LAB data are calculated using manually labeled data, and are thus guaranteed to be more accurate.

We expected that training with WILD data would improve results on the WILD data because the training and testing distributions are similar. However, our performance in the WILD/WILD experiment (Figure 10b) is worse than in the LAB/WILD experiment (Figure 10a). This reduction could be explained by the fact that training on LAB data was done with accurate labels, whereas only weak labels are available for WILD data. Therefore, we also trained on LAB data with synthetically generated weak labels (Figure 10b). Nonetheless, training on weakly labeled LAB data still outperforms training on WILD data for all participants except participant 11. Combining the weakLAB and WILD data during training does not lead to significant differences in performance from training on only WILD data, likely due to the fact that we have six times more WILD data than LAB data.

The drop in performance from training on WILD data versus LAB data is different from Hammerla et al.’s findings: wWth their dataset of 34 participants, training on WILD data led to higher performance on WILD data than training on LAB data [7]. Our results may be a reflection of the discrepancy [42,43] of WILD data labels obtained from different participants. It is possible that Hammerla et al. [7] were able to bypass the issues of labeler discrepancy with their stratified seven-fold cross-validation method. Mixing data from all participants allowed their system to learn each participant’s movement and labeling style. It could therefore benefit from the greater variability in the WILD data. Zhang et al. [5] also showed improved performance when training on WILD, but the algorithms were trained on person-specific data. In contrast, our LOSO cross-validation method does not allow our system to learn these idiosyncrasies. Our performance may therefore suffer from the discrepancy and inaccuracies of the WILD data labels.

### 4.4. Estimating Percentage of Tremor Time

Most of the previous experiments have evaluated the accuracy of various systems in detecting exactly when tremor occurred. However, an aggregate percentage of tremor time over long periods has been used in PD neurological studies to determine differences in cortical activation patterns [44] or to analyze the oscillatory activity and correlations throughout the different states of levodopa-naive Parkinsonism [45].

**Figure 11:**
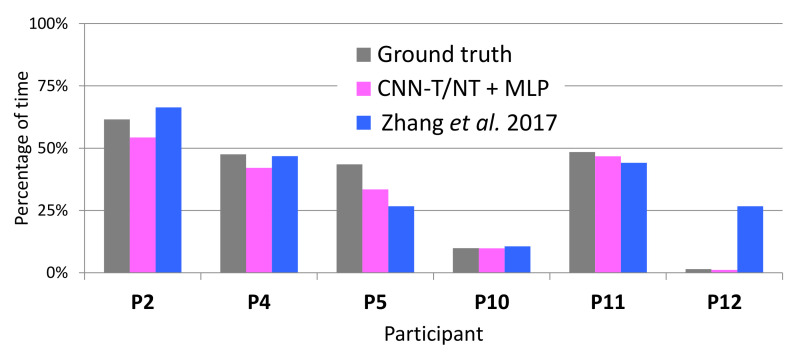
True percentage of tremor (calculated from the accurate labels) and percentage output by the automatic systems on LAB data.

Figure 11 shows the ground truth percentage of tremor time, which was computed from the accurate labels of the LAB data, and compares it to the percentage output by CNN-T/NT + MLP, MFCCs-T/NT + MLP, and Zhang et al. [4]. The percentage of tremor time was computed during the whole session. Table 1 show the sessions’ durations per participant in seconds (between 3090 and 5824 seconds). With the exception of participant 4, the error between CNN-T/NT + MLP or MFCCs-T/NT + MLP and ground truth is on par with or less than Zhang et al. [4].

**Figure 12:**
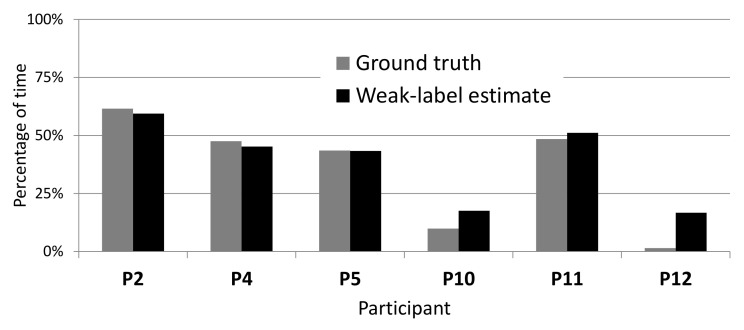
Comparing true percentage of tremor to percentage estimated from weak labels on LAB data.

On WILD data, accurate labels are not available, and each weak label corresponds to a range of tremor percentage: 0–33%, 33–66%, and 66–100% tremor for *Almost none*, *Half the time*, and *Almost always*, respectively. The estimate of the percentage of tremor time from these labels corresponds to the middle of the range (i.e., 16.6%, 50%, and 83.3% tremor, respectively). The percentage of tremor time was computed using all the data of each participant, around 300 entries of five minutes (25 h). Figure 12 shows how these estimates using weak labels compare to the true percentage on LAB data. Although our weak-label estimates generally correspond well to the ground truth, they overestimate the true percentage on participants 10 and 12, most likely because these participants experienced very little tremor.

**Figure 13:**
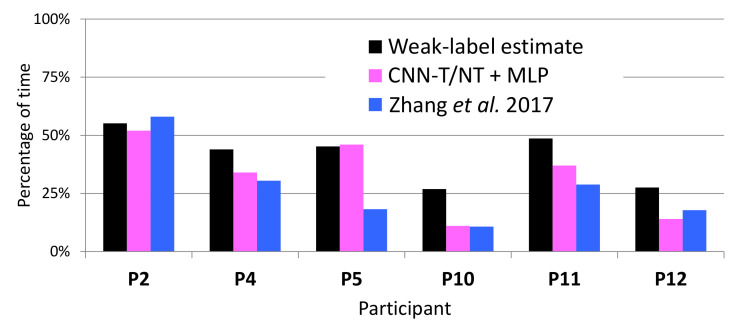
Weak-label estimate of percentage of tremor compared to percentages output by the automatic systems on WILD data.

Figure 13 compares the percentage of tremor time detected by the systems to that estimated from the weak labels on WILD data. Consistently with our findings on the LAB data, Figure 13 indicates that CNN-T/NT + MLP and MFCCs-T/NT + MLP have less error (with respect to the weak-label estimates of percentage) than Zhang et al. [4]. Both systems reported a lower percentage of tremor time for participants 10 and 12 than the weak-label estimate, which may be a reflection of the findings from Figure 12: weak-label estimates of percentage of tremor time are too high for participants 10 and 12. Note that, on WILD data, the weak-label estimates may deviate even further from the ground truth than what is indicated in Figure 12 due to the fact that weak labels obtained from WILD data are more noisy than those computed from LAB data.

**Figure 14:**
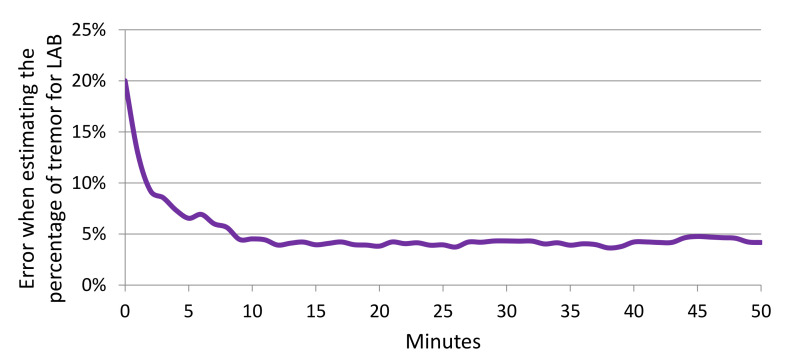
Error when estimating the percentage of tremor depending on the duration (minutes) considered to estimate the percentage.

Table 5 shows the mean and standard deviation of the error when estimating the percentage of tremor for LAB and WILD (using data from Figure 11 and Figure 13). In all scenarios, the proposals described in this paper (CNN-T/NT + MLP and MFCCs-T/NT + MLP) show improvement over Zhang et al. [4]. For LAB data, the percentage of tremor time was computed with the whole session. Figure 14 shows the error across subjects when predicting the percentage of tremor time with smaller periods of time (minutes) using LAB data. The error decreases rapidly when increasing the period time, that is, with periods of time between 10 and 15 min, it is possible to obtain a similar error compared to the case of considering the whole session. As we have only in-the-wild recordings from six patients, the differences between the three evaluated methods are not statistically significant (*t*-test *p*-value > 0.3). Although there are not significant differences between methods, the mean error when estimating the percentage of tremor is lower than 5% for LAB data and lower than 10% for WILD data. Recent studies proposing the percentage of tremor as a PD biomarker to provide continuous monitoring of tremor have reported errors of over 15% [46].

## 5. Conclusions

This paper analyzes several algorithms and features for PD tremor detection in laboratory and in-the-wild data using wrist-worn accelerometers. In particular, we have identified several feature sets that demonstrate improvement over previous work, including a tremor spectrum extraction technique. We found that CNN-T/NT + MLP leads to the highest performance among the twelve systems we analyzed, and that it significantly outperforms the three most relevant systems described in the literature with sufficient detail to be reproduced. Improved performance of CNN-T/NT over CNN indicates the benefit of our novel tremor spectrum extraction technique. Our handcrafted MFCCs-T/NT features have similar performance to the CNN-T/NT features, and are shown to be less sensitive to lack of training data than the CNN-T/NT. When developing a real system, we suggest using MFCCs-T/NT when the amount of available data is not sufficient to train a deep neural network. In this situation, handcrafted features with traditional machine learning algorithms, like Random Forest, provide a reasonable performance. Additionally, the method for tremor spectrum extraction proposed in this paper contributes to increase this performance independently of the amount of available data. On the other hand, if there are enough data for training, the CNN-T/NT approach is preferred because this approach can learn relevant features for tremor detection.

We analyzed the ability of our best system (CNN-T/NT + MLP) and the best system from previous work (Zhang et al. [4]) to reproduce in-home tremor self-assessments. Our system performs better than that of Zhang et al. [4], although the improvement is not statistically significant. Our findings do not coincide with those of Hammerla et al. [7] or Zhang et al. [5], i.e., performance degrades when trained on WILD data as opposed to LAB data, even when controlling for weak versus accurate labels or combining WILD with LAB data. The discrepancy in our findings may be due to our differing data splitting procedures (LOSO versus seven-fold and person-specific). In this paper, we have used LOSO, guaranteeing that there are not recordings from the same subject in training and testing sets. From our point of view, the LOSO methodology simulates a more realistic scenario where there are no training labels for a new patient. Using a seven-fold cross-validation methodology can guide us to optimistic conclusions that are not applicable when developing a real system.

We also compared several systems on their ability to estimate a percentage of tremor time over long time periods, which has been used in previous neurological studies to analyze the PD’s impact in different patients. CNN-T/NT + MLP performs better than Zhang et al. [4], with an average error of 4.1% when predicting percentage of tremor time on LAB data, and 9.1% on WILD data. Note that, on the WILD data, percentage of tremor time could only be estimated from the weak labels, and is thus not a perfect ground truth. These results improved the performance described in a recent study that proposed the percentage of tremor as a biomarker for PD monitoring, reporting estimation errors of over 15%. This recent study also used acceleration signals from sensors situated in a wristband.

The main contributions of this paper comparing with the best system from previous work (Zhang et al. [4]) have been the analysis of several features sets, including proposals adapted from the area of audio processing, the evaluation of deep learning algorithms, and the description of a signal preprocessing step to extract tremor information from the signal spectrum.

While the proposed features provided better results in the evaluated data, a limitation of this work is the small number of subjects. However, we have partially mitigated the small sample-subject size by using an LOSO cross-validation methodology. Another limitation relevant for the monitoring of PD progression is that we only detect presence/absence of tremor, but not the intensity or type of tremor (resting, postural, or kinetic). While future work might address previous limitations, a major challenge continues to be to improve the quantity and quality of labels in the wild. For instance, personalization algorithms that can improve detection scores from unlabeled user-specific data can lead to transformative results towards improving generalization of the detection algorithms. Along these lines, a framework that can simultaneously train on accurate (LAB) and weak (WILD) labels could help leverage the high variability we find in WILD data, while reducing the effect of poor label quality. Another interesting future line will consist of using the proposed methods to supervise tremor of PD patients before and after Levodopa therapy. This analyses will allow the obtaining of objective metrics (biomarkers) to quantify the medication impact. Evaluating the system with already validated therapies would support the feasibility of the proposed system.

## Figures and Tables

**Table 1:** Laboratory session duration (s) and percentage of tremor time for left and right hands.

Participant	Laboratory Session Duration (s)	Left Hand	Right Hand
2	4495	80%	41%
3	3351	59%	74%
4	3314	57%	37%
5	5284	39%	44%
7	5824	27%	19%
8	5478	9%	38%
9	5766	22%	8%
10	5070	8%	12%
11	3090	69%	26%
12	4480	2%	1%

**Table 2:** Unified Parkinson’s Disease Rating Scale (UPDRS) evaluations (provided by a medical expert).

Participant #	MDS-UPDRS Task											
	Resting		Postural		Kinetic		Finger		Hand		Pron.	
	Tremor		Tremor		Tremor		Tapping		Mov		Sup.	
	(3.17)		(3.15)		(3.16)		(3.4)		(3.5)		(3.6)	
	L	R	L	R	L	R	L	R	L	R	L	R
2	2	2	2	1	1	1	1	1	1	0	3	0
3	1	2	1	2	1	2	1	3	1	2	2	2
4	3	3	2	3	1	1	3	1	3	1	4	3
5	0	0	2	2	2	2	2	1	2	1	2	2
7	0	0	1	1	1	1	1	3	2	3	3	2
8	1	1	0	1	1	1	1	2	1	2	1	2
9	0	0	0	0	0	0	3	2	-	2	-	4
10	0	2	0	0	1	0	2	3	1	1	2	1
11	3	0	3	0	1	0	3	1	1	0	1	0
12	1	1	0	0	0	0	3	2	3	2	2	2

Note: The arm of participant 9 was rigid. Therefore, the hand movement and pronation–supination tasks were skipped.

**Table 3:** Area Under the Curve (AUC) and False Positive Rate (FPR) at a 0.90 True Positive Rate (TPR) for the seven feature sets and two classifiers: Random Forest (RF) and Multi-Layer Perceptron (MLP).

Feature Set	AUC		FPR at 0.90 TPR	
	RF	MLP	RF	MLP
Energy threshold	0.715		0.62	
PSD	0.813	0.818	0.53	0.52
Baseline	0.830	0.829	0.45	0.45
MFCCs	0.851	0.853	0.40	0.39
CNN	0.850	0.850	0.38	0.41
MFCCs-T/NT	0.869	0.870	0.33	0.33
CNN-T/NT	0.884	0.887	0.32	0.30

Note: The energy threshold constitutes a 1D feature vector, which makes RF and MLP unapplicable. AUC and FPR at a 0.90 TPR are computed by sliding the threshold over the range of possible values.

**Table 4:** AUC and FPR at 0.90 TPR on LAB data

System	AUC	FPR at 0.90 TPR
CNN-T/NT + MLP (ours)	0.887	0.30
Pulliam et al [30]	0.701	0.67
Hammerla et al. [7]/Fisher et al. [32]	0.809	0.50
Zhang et al. [4]	0.831	0.44

**Table 5:** Mean and standard deviation of the error when estimating the percentage of tremor for LAB and WILD.

System	LAB Data		WILD Data	
	Mean	Std	Mean	Std
CNN-T/NT + MLP (ours)	4.1%	4.0%	9.1%	5.9%
MFCCs-T/NT + MLP (ours)	4.4%	5.4%	12.1%	8.2%
Zhang et al. [4]	8.8%	10.0%	13.7%	10.1%

Note: Errors on WILD data are computed with respect to percentages of tremor that are estimated from weak labels.
